# Functional conservation of the Drosophila hybrid incompatibility gene *Lhr*

**DOI:** 10.1186/1471-2148-11-57

**Published:** 2011-03-02

**Authors:** Nicholas J Brideau, Daniel A Barbash

**Affiliations:** 1 Department of Molecular Biology and Genetics, Cornell University, Ithaca, NY 14850, USA

## Abstract

**Background:**

Hybrid incompatibilities such as sterility and lethality are commonly modeled as being caused by interactions between two genes, each of which has diverged separately in one of the hybridizing lineages. The gene *Lethal hybrid rescue *(*Lhr*) encodes a rapidly evolving heterochromatin protein that causes lethality of hybrid males in crosses between *Drosophila melanogaster *females and *D. simulans *males. Previous genetic analyses showed that hybrid lethality is caused by *D. simulans Lhr *but not by *D. melanogaster Lhr*, confirming a critical prediction of asymmetry in the evolution of a hybrid incompatibility gene.

**Results:**

Here we have examined the functional properties of *Lhr *orthologs from multiple Drosophila species, including interactions with other heterochromatin proteins, localization to heterochromatin, and ability to complement hybrid rescue in *D. melanogaster*/*D. simulans *hybrids. We find that these properties are conserved among most *Lhr *orthologs, including *Lhr *from *D. melanogaster*, *D. simulans *and the outgroup species *D. yakuba*.

**Conclusions:**

We conclude that evolution of the hybrid lethality properties of *Lhr *between *D. melanogaster *and *D. simulans *did not involve extensive loss or gain of functions associated with protein interactions or localization to heterochromatin.

## Background

The Biological Species Concept is a widely accepted definition of species in which species are defined as groups of interbreeding populations that are reproductively isolated from other groups [1]. Hybrids between closely related species are often inviable or sterile, and this inviability and sterility is collectively called hybrid incompatibility (HI). Crosses between *Drosophila melanogaster *and its sibling species *D. simulans *produce incompatible hybrids. In the cross of *D. melanogaster *females to *D. simulans *males, the F1 daughters are semi-viable but sterile, and the sons are inviable [2].

Two genes, *Hybrid male rescue (Hmr) *in *D. melanogaster*, and *Lethal hybrid rescue *(*Lhr*) in *D. simulans*, are major-effect contributors to hybrid incompatibility on the basis that loss-of-function mutations in either gene can suppress hybrid male lethality [3-6]. *Lhr *encodes a small protein (319 AA) and appears to be *Drosophila *specific, as we are unable to identify homologs in vertebrates or in other invertebrates, including in other insect genera [5]. Furthermore, among *Drosophila *species the coding sequence of *Lhr *is also highly divergent, with limited regions of identity as well as extensive indel variation. For example, *D. melanogaster *LHR shares 80, 71, and 39 percent protein identity with *D. simulans*, *D. yakuba *and *D. virilis *LHR orthologs, respectively. Between *D. melanogaster *and *D. simulans*, *Lhr *has a high K_A_/K_S _value of 0.731, and population genetic analyses demonstrated that this high divergence is consistent with positive selection [5].

LHR localizes to heterochromatic sites on salivary gland polytene chromosomes in a pattern similar to, but not completely overlapping with, Heterochromatin Protein 1 (HP1) [5]. HP1 is a well-studied chromosomal protein that is found in eukaryotes ranging from fission yeast to humans [7], and is encoded by the *Su(var)2-5 *gene in *Drosophila *[8]. HP1 localizes to pericentric and telomeric heterochromatin, and to approximately 200 euchromatic sites throughout the genome [9,10]. The localization of HP1 is consistent with its multiple functions in establishment and spreading of heterochromatin, telomere capping, and both silencing and activation of gene expression [11-13].

Greil et al. [14] identified four new heterochromatin proteins (HPs) based on their association with HP1, which they named HP3, HP4, HP5 and HP6. HP3 was independently identified as LHR [5]; HP4 was independently identified as the HP1-interacting protein (Hip) [15]; and HP6 was independently identified as the protein Umbrea [16,17]. Like mutations in HP1, mutations in HP4 and HP5 dominantly suppress position-effect variegation, and analysis of HP6 suggests that it has a role in telomere protection [14-16]. HP3 (LHR), HP4 (Hip) and HP5 all have similar expression patterns as HP1, being enriched in ovaries and early embryos [18]. Based on their common localization to heterochromatin, their similar expression patterns, and their proposed physical interactions, HP1, LHR, HP4, and HP5 may form a multi-protein heterochromatic complex *in vivo*.

Of particular interest is whether and how the interactions and functions of LHR may be evolving. This interest stems from a fundamental prediction of the Dobzhansky-Muller model that HIs are asymmetric [19]. Here this means that hybrid male lethality is caused specifically by *D. simulans *LHR (sim-LHR), and not by *D. melanogaster *LHR (mel-LHR). We previously confirmed this prediction of asymmetry by demonstrating that a loss-of-function mutation in *sim-Lhr *suppresses hybrid male lethality, but loss-of-function deletions of *mel-Lhr *do not [5]. Asymmetry of hybrid lethality properties has also been experimentally demonstrated for the gene *Hmr *[20].

What is the nature of this functional divergence for an HI protein like LHR, especially in light of the evidence that LHR is rapidly evolving and has diverged under adaptive evolution? One possibility is that sim-LHR or mel-LHR has evolved a new function (neofunctionalization) distinct from their common ancestor. Another possibility is that sim-LHR or mel-LHR has lost ancestral functions. Finally, one could envision that divergence has led to complex or subtle changes in function.

We showed previously that sim-LHR and mel-LHR share the ability to interact with mel-HP1 in a yeast two-hybrid assay [5], suggesting that LHR has not undergone a complete change in functional properties between *D. melanogaster *and *D. simulans*. Here we have examined several properties of mel-LHR, including interactions with other HPs and localization to the chromocenter. To potentially discover differences among LHR orthologs, we have specifically addressed whether the localization and interaction properties of mel-LHR are conserved for other LHR orthologs, and have tested for the ability to induce hybrid male lethality. Surprisingly, we find that all LHR orthologs tested appear to have the same molecular interaction and localization properties, and most strikingly, three have the same ability to induce *D. melanogaster-D. simulans *hybrid male lethality.

## Results

### Variable rates of HP gene evolution

Four heterochromatin proteins have been characterized and named (HP3-HP6) based on their association with HP1 [14]. Interestingly, these HP1 interacting proteins have two distinct patterns of molecular evolution (Table 1; Additional file 1: Supplemental Figure S1). Both HP1 and HP4 are well conserved between *D. melanogaster *and *D. simulans *with low K_A_/K_S _values that are typical of genes evolving under stabilizing selection, while LHR (HP3), HP5, and HP6 have high K_A_/K_S _values that suggest either adaptive evolution or relaxed constraint. The different patterns of molecular evolution are intriguing given that several of these HPs interact with one another, and all interact with the well-conserved HP1 (Additional file 1: Supplemental Figure S1). We were therefore interested to explore further the relationships between LHR and these four other HPs.

**Table 1:** Divergence of heterochromatin proteins (HPs) between *D. melanogaster *and D. simulans

Gene	K_a_	K_s_	K_a_/K_s_
*Su(var)2-5 *(*HP1*)	0.017	0.115	0.135
*Lhr *(*HP3*)*	0.078	0.106	0.731
*HP4 *(*Hip*)	0.017	0.121	0.127
*HP5*	0.079	0.100	0.790
*HP6 *(*Umbrea*)	0.132	0.133	0.988

*- values are from ref. [5].

### LHR binds directly to HP1

**Figure 1:**
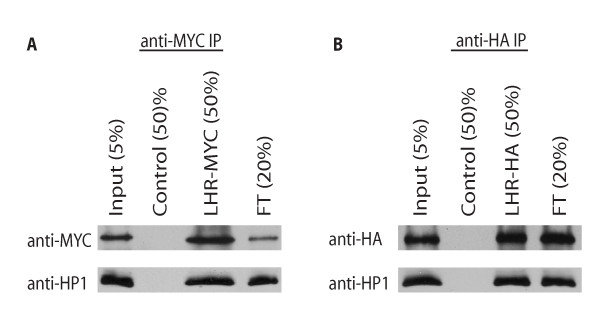
**HP1 immunoprecipitates with and binds directly to LHR**. A) HP1 immunoprecipitates with LHR in extracts from *Drosophila *Kc embryonic tissue culture cells. Anti-MYC immunoprecipitates from wild-type Kc cells (control) and LHR-MYC expressing cells were analyzed by Western blot using anti-MYC and anti-HP1 antibodies. The percent of each sample loaded is shown (%). Protein that did not immunoprecipitate is shown in the flow through lane (FT). B) LHR binds to HP1 in an *in vitro *binding assay. *In vitro *synthesized LHR-HA and HP1 proteins were mixed and the resulting complexes were immunoprecipitated with anti-HA antibody and analyzed by Western blot using anti-HA and anti-HP1 antibodies. Control lane is sample immunoprecipitated from an *in vitro *synthesis extract without DNA from either gene added.

LHR has been shown to interact in yeast two-hybrid assays with HP1 and HP6 [5,21]. Because HP1 is required to localize LHR (see below), we wanted to determine whether these proteins interact directly. We therefore performed immunoprecipitation assays with extracts from two sources. First, we used nuclear extracts from *Drosophila *Kc cells that were transiently transfected with a LHR-MYC fusion construct. We found that endogenously expressed HP1 immunoprecipitates with MYC-tagged LHR (Figure 1A).

It is possible, however, that LHR and HP1 co-associate by each binding to another protein rather than directly binding to each other. Therefore, to test if LHR can bind directly to HP1, we performed an *in vitro *binding assay. We found that *in vitro *synthesized LHR-HA and HP1 also co-immunoprecipitate (Figure 1B). In combination with the published yeast two-hybrid and immunofluorescence data, our *in vitro *results strongly suggest that LHR and HP1 co-localize in heterochromatin due to direct physical binding to each other.

Because it has been suggested that LHR is part of a network of HP1-associated proteins [14] we used yeast two-hybrid to test if LHR interacts with HP4, HP5 and HP6. Of these three other HPs we examined, only HP6 interacts with LHR (see Figure 4 below and data not shown), consistent with previously reported results [21].

### Mapping the interacting regions of HP1 and LHR

**Figure 2:**
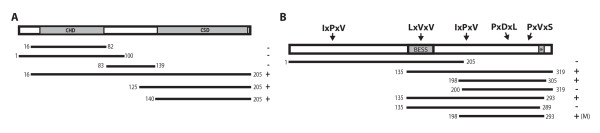
**Identifying the interacting domains in HP1 and LHR**. A) The chromo shadow domain of HP1 interacts with LHR. Full length *D. melanogaster *HP1 is shown with the chromo domain (CHD) and chromo-shadow domain (CSD) shaded. Fragments of HP1 that were tested for interaction with LHR in a yeast two-hybrid assay are shown below. The symbols on the right indicate non-interacting (-) and interacting (+) fragments. B) A 96 AA minimal interacting fragment (M), inferred from the LHR fragments tested, interacts with HP1. Full length LHR is shown with the BESS domain and predicted NLS (*) shaded. The locations of the three amino acids for each of the five pentamer motifs in LHR that were converted to alanine by site-directed mutagenesis and individually tested for interaction with HP1 are shown (top). None of the single-motif mutants abolished interaction with HP1 in yeast two-hybrid assays. Fragments of LHR that were tested for interaction with HP1 in a yeast two-hybrid assay are shown below.

HP1 has multiple functions that are mediated by different domains [17,22]. To define which region of HP1 is responsible for interacting with LHR, we created a series of derivatives of HP1 and tested them in a yeast two-hybrid assay. We found that the C-terminal chromo-shadow domain (CSD) is both necessary and sufficient to interact with full length LHR (Figure 2A). This result is consistent with other findings showing that the CSD of HP1 mediates protein-protein interactions [15,23]. Since HP6 also interacts with LHR and has a chromo-shadow domain similar to HP1, it is possible that LHR simply interacts with any protein containing a CSD. This explanation is unlikely, however, because we observed no interaction between LHR and the *D. melanogaster *HP1 paralog Rhino, which also contains a CSD (Additional file 1: Supplemental Figure S2).

Many proteins that interact with the chromo-shadow domain of HP1 contain either the consensus peptide pentamer PxVxL, or variations of this motif [24,25]. We were unable to detect any canonical HP1-binding motifs in *D. melanogaster *LHR, but did find five potential PxVxL variants (Figure 2B). However, mutagenesis of each motif demonstrated that none of the 5 motifs are individually essential for the HP1 interaction (Additional file 1: Supplemental Figure S3).

We therefore mapped the HP1 interaction domain in LHR by testing a series of LHR fragments for interaction in yeast two-hybrid assays. By analyzing the interacting and non-interacting two-hybrid fragments, we inferred that the smallest HP1-interacting fragment of LHR contains 96 amino acids and is located in the C-terminal half of the protein (labeled "M" in Figure 2B).

### Heterochromatic localization of LHR depends on HP1 in Drosophila

**Figure 3:**
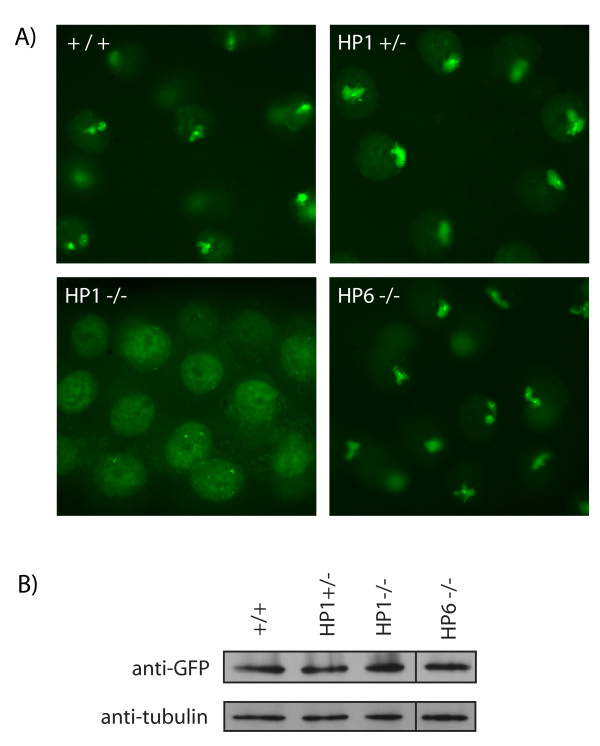
**LHR-YFP localization depends on HP1**. A) Live YFP analysis of LHR-YFP in whole-mount salivary glands from third instar larvae in wild-type and HP-mutant backgrounds. A recombinant chromosome containing *P{UAS-mel\Lhr::Yfp}* and a salivary gland specific GAL4 driver was crossed into HP mutant backgrounds. The bright heterochromatic foci normally seen in wild type animals are absent in *HP1* mutants but not in *HP6* mutants. B) LHR-YFP is expressed and stable in HP mutants. Extracts from third-instar larval salivary glands were loaded on an SDS-PAGE gel and analyzed by Western blot using anti-GFP and anti-tubulin antibodies. Relative to the tubulin loading controls, LHR-YFP is present at similar levels in all samples analyzed.

LHR was previously shown to mislocalize in Drosophila embryonic tissue-culture cells when HP1 is knocked down by RNAi [14,26]. We created *Lhr *transgenes in order to confirm this result in whole animals and to develop an assay system for examining *Lhr *ortholog evolution. We made a fusion of mel-LHR to Yfp (termed *mel\Lhr::Yfp*) under control of the GAL4/UAS expression system, and generated transgenic flies that carry this *UAS-mel\Lhr::Yfp *transgene. We used a salivary-gland specific GAL4 driver and examined the localization of LHR by live YFP analysis or anti-GFP staining in fixed tissues. We found that LHR-YFP loses heterochromatic targeting and is redistributed throughout the nucleus in *HP1 *mutant larvae (Figure 3A). It is possible that the heterochromatic localization of LHR-YFP is lost in the *HP1 *mutant because the protein is unstable. Western blots indicate, however, that the protein is present at a similar level in the mutant HP1 background as in the wild type background (Figure 3B). In contrast to these results with *HP1 *mutants, we did not observe any localization differences for LHR-YFP in salivary glands from *HP6 *mutant animals (Figure 3A).

### LHR orthologs interact with HP1 and HP6

**Figure 4:**
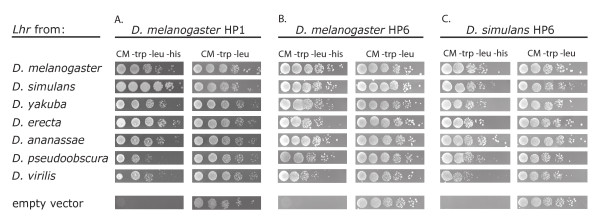
**Seven LHR orthologs interact with HP1 and HP6**. A) LHR from seven Drosophila species interacts with *D. melanogaster* HP1 in a yeast two-hybrid assay. Interactions were detected by activation of HIS3 and growth on media lacking histidine (CM-trp-leu-his); growth controls (CM-trp-leu) contain histidine. The same seven LHR orthologs also interact with (B) *D. melanogaster* HP6 and (C) *D. simulans* HP6.

To test if the property of HP1 interaction is conserved among LHR orthologs we cloned the coding sequences from seven species and found that all of them interact with *D. melanogaster *HP1 in a yeast two-hybrid assay (Figure 4A). We then tested whether these LHR orthologs can also interact with *D. melanogaster *and *D. simulans *HP6, because HP6 has a much higher rate of divergence between *D. melanogaster *and *D. simulans *than HP1 (Table 1). We found that all seven LHR orthologs tested interact with both mel-HP6 and sim-HP6 (Figure 4B,C). These data demonstrate that LHR interactions with heterochromatin proteins discovered in *D. melanogaster *are likely to be conserved among *Drosophila *despite highly variable rates of evolution of these proteins, especially the C-terminal region of LHR (Additional file 1: Supplemental Figure S4).

### LHR orthologs localize to heterochromatin when expressed in *D. melanogaster*

We have demonstrated that LHR from all species tested can interact with *D. melanogaster *HP1, and that HP1 is required in *D. melanogaster *for LHR to localize to heterochromatin. Other studies in *D. melanogaster*, however, suggest that LHR localizes to some sites by an unknown mechanism, independently of HP1 [14,26]. Therefore, we wished to test if other *Drosophila *LHR orthologs would also localize to heterochromatin. Because of the difficulty in directly determining LHR localization in the absence of an antibody recognizing LHR orthologs, we developed an assay system for localization of LHR orthologs expressed in *D. melanogaster*. We tested LHR orthologs from *D. simulans*, *D. yakuba*, and *D. virilis*, which together represent a range of divergence from *D. melanogaster *LHR.

We transformed UAS-driven LHR-YFP fusion constructs from these species into a common *attP *site on chromosome 3 of *D. melanogaster *using the Φ C31 site-specific integration system [27]. Using this system, transgenes can be inserted into the same position in the genome, allowing a direct comparison between *Lhr *orthologs without complications from variation due to position effects on transgene expression. We were unable to obtain a transformant of *D. melanogaster *LHR-YFP into the same *attP *site, so as an alternative, we used P element transformation and obtained three independent transgene insertion lines.

**Figure 5:**
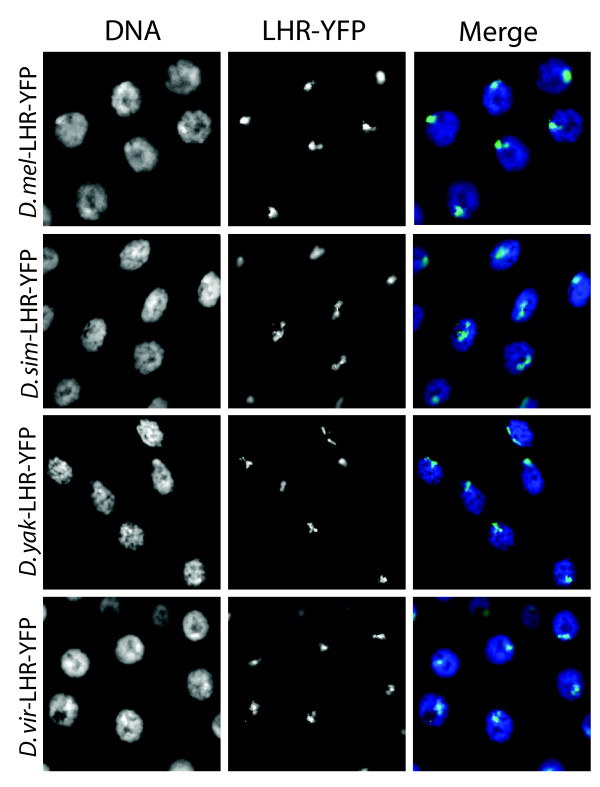
**Four LHR orthologs localize to similar foci when expressed in *D. melanogaster***. Live YFP analysis in whole-mount salivary glands from third instar larvae expressing *Lhr-Yfp* from *D. melanogaster*, *D. simulans*, *D. yakuba*, and *D. virilis*. For each *Lhr* ortholog tested, *D. melanogaster* females carrying the *Lhr-Yfp *transgene were crossed to males carrying a salivary-gland specific *GAL4* driver, and the F1 heterozygous larvae were analyzed. DNA was stained using DAPI.

**Figure 6:**
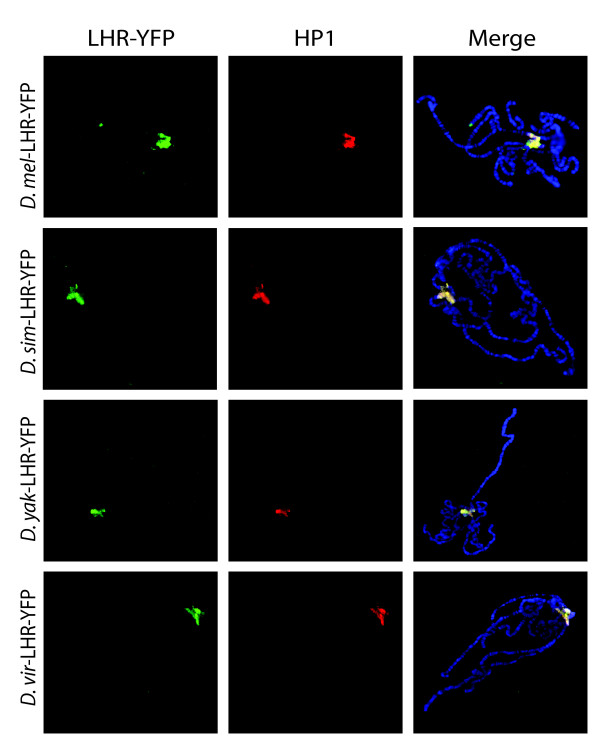
**Four LHR orthologs co-localize with HP1 at the chromocenter**. Salivary gland polytene chromosome squashes co-stained with anti-GFP (green), anti-HP1 (red) and DAPI (blue in merge). The same crosses were used as in Figure 5.

Live YFP analysis in whole mount salivary glands showed similar bright foci for all three LHR orthologs when compared to *D. melanogaster *LHR-YFP (Figure 5). Anti-GFP staining in polytene squashes demonstrated that the foci observed in the whole mount tissues for all four LHR orthologs overlap with the pericentric heterochromatin at the chromocenter. We also co-stained for HP1 and found that all four LHR orthologs co-localize with *D. melanogaster *HP1 in heterochromatin when expressed in *D. melanogaster *(Figure 6). These results demonstrate that the property of heterochromatin localization is likely conserved among LHR orthologs.

### Three *Lhr *orthologs have hybrid lethal activity

The above results suggest that LHR has not likely evolved different interaction or localization properties between *D. melanogaster *and *D. simulans*, or among outgroup species. These findings raise the question of whether the hybrid lethality activity of *sim-Lhr *is unique. We tested this question using the genetic assay previously developed to identify the *sim-Lhr *gene [5]. We crossed *D. melanogaster *females heterozygous for the different *UAS *driven *Lhr-Yfp *transgenes and the *Actin5C-GAL4 *driver to *D. simulans Lhr*^1 ^males, and scored the hybrid male progeny (Additional file 1: Supplemental Figure S5). In these crosses, we are testing whether the ubiquitous *Lhr *expression from the *Actin5C-GAL4 *driver can suppress the hybrid male rescue from the loss-of-function *D. simulans Lhr^1 ^*mutation.

We first performed control crosses to *D. melanogaster*. *P{UAS-mel\Lhr::Yfp}1 *and *P{UAS-mel\Lhr::Yfp}2 *will segregate independently from the *GAL4 *transgene (Table 2; Additional file 1: Supplemental Figure S5). In the absence of viability effects one expects a 2:1:1 ratio of eye colors, which we observed. *P{UAS-mel\Lhr::Yfp}3 *is on chromosome 3 and was thus trans-heterozygous to the *GAL4 *transgene in the mothers of the crosses. Progeny ratios in the control cross again did not deviate significantly from the expected ratios, demonstrating that the *P{UAS-mel\Lhr::Yfp}3 *transgene is unlinked to the *GAL4 *transgene and that its expression has no affect in *D. melanogaster*. Similar results were obtained in control crosses with the *Lhr-Yfp *transgenes from *D. simulans*, *D. yakuba *and *D. virilis *(Table 2). We conclude that expression of these four *Lhr *orthologs does not affect viability of *D. melanogaster*. In experimental crosses where *UAS*-*sim\Lhr::Yfp *was crossed to the *D. simulans Lhr*^1 ^strain to generate hybrids, the progeny ratios differed significantly from those in the control cross. This difference reflects suppression of hybrid male rescue by *Lhr*^1 ^as judged by the presence of approximately half of the *GAL4*-containing sons (red-eyed class) compared to the control cross (Table 2). This result is similar to what we previously observed with untagged *sim-Lhr *transgenes [5] and thus demonstrates that the *Yfp *tag does not alter *Lhr *function. We also observed here a deficit in *UAS/+ *progeny compared to their *+/+ *siblings, which we also saw previously and attributed to maternally inherited GAL4 protein activating expression of the zygotically inherited *UAS *transgene.

**Table 2:** Testing if four *Lhr *orthologs have hybrid male lethal activity

	F1 Progeny Class		Number of progeny	
*UAS *transgene in *D. melanogaster *mother *^a^*	Phenotype	Genotype	*D. mel *control cross *^b^*	***D. sim Lhr***^***1 ***^**cross **^***c***^
*P{UAS-mel\Lhr::Yfp}1*	Red-eyed male	UAS/+; GAL4/+ and	278	134
		+/+; GAL4/+		
	Orange-eyed male	UAS/+; +/+	148	107
	White-eyed male	+/+; +/+	143	141
			
		Total	569 ^n.s.^	382***
				
*P{UAS-mel\Lhr::Yfp}2*	Red-eyed male	UAS/+; GAL4/+ and	221	334
		+/+; GAL4/+		
	Orange-eyed male	UAS/+; +/+	93	8
	White-eyed male	+/+; +/+	102	314
			
		Total	416 ^n.s.^	656***
				
*P{UAS-mel\Lhr::Yfp}3*	Red-eyed male	UAS/+; GAL4/+ and	282	74
		+/+; GAL4/+		
	Orange-eyed male	UAS/+; +/+	162	2
	White-eyed male	+/+; +/+	175	82
			
		Total	619 ^n.s.^	158***
				
*Φ {UAS-sim\Lhr::Yfp*}	Red-eyed male	UAS/+; GAL4/+ and	139	117
		+/+; GAL4/+		
	Orange-eyed male	UAS/+; +/+	72	39
	White-eyed male	+/+; +/+	77	107
			
		Total	288 ^n.s.^	223***
				
*Φ {UAS-yak\Lhr::Yfp*}	Red-eyed male	UAS/+; GAL4/+ and	83	346
		+/+; GAL4/+		
	Orange-eyed male	UAS/+; +/+	37	55
	White-eyed male	+/+; +/+	45	396
			
		Total	165 ^n.s.^	797***
				
*Φ {UAS-vir\Lhr::Yfp*}	Red-eyed male	UAS/+; GAL4/+ and	158	164
		+/+; GAL4/+		
	Orange-eyed male	UAS/+; +/+	76	114
	White-eyed male	+/+; +/+	86	112
			
		Total	320 ^n.s.^	390 ^n.s.^

*^a ^*- Females heterozygous for the indicated *UAS *transgene and for *P{Act5C-GAL4}17bFO1 *were crossed to either *D. melanogaster w^1118^/Y *(control cross) or to *D. simulans Lhr^1 ^*males. For the first two sets of crosses the females were of genotype *w/w; UAS/+; GAL4/+*; for the remaining crosses the females were of genotype *w/w; UAS +/GAL4 +*.

*^b ^*- For the control crosses the ratios of red eye: orange eye: white eye progeny were tested for deviation from a 2:1:1 ratio using a chi-square test. n.s. = p > 0.05

*^c ^*- The ratios of red eye: orange eye: white eye for hybrid crosses were compared to control crosses using a 3 × 2 contingency table and chi-square test. *** = p < 1 × 10^-3^; n.s. = p > 0.05, 2 degrees of freedom.

Surprisingly, progeny in experimental crosses with *Lhr-Yfp *transgenes from *D. melanogaster *and *D. yakuba *also differed significantly from their respective control crosses. We confirmed by PCR that the rescued red-eyed males only carried the *GAL4 *transgene (n = 10 for each cross), thus concluding that the reduction in red-eyed males is caused by lethality of sons inheriting both the *GAL4 *and *UAS-Lhr-Yfp *transgenes. In two of the three crosses with *UAS-mel\Lhr::Yfp *and in the cross with *UAS-yak\Lhr::Yfp *we also again observed a strong reduction in the viability of *UAS/+ *(orange-eyed) sons. These results demonstrate that the hybrid lethality activity of *Lhr *is not unique to *D. simulans Lhr*, at least when expressed under control of *actin-Gal4*. Experimental crosses with the *D. virilis *transgene had a slight reduction in the number of red-eyed males, but this cross did not differ significantly from the controls. This result suggests that *vir-Lhr *does not induce lethality in *D. melanogaster*-*D. simulans *hybrids.

## Discussion

### LHR interaction and localization properties

Giot et al. [21] reported that LHR interacts with HP1 and HP6 in a large-scale yeast two-hybrid screen. We previously confirmed this result for HP1, and have done so here for HP6. We have further shown here that HP1 immunoprecipitates with LHR in cell culture extracts, and that LHR binds to HP1 in an *in vitro *assay. These data indicate that the interaction between the LHR and HP1 proteins is likely to be direct. We also discovered that LHR interacts with the chromo-shadow domain of HP1. Unlike many other CSD-interacting proteins [28], we did not find a PxVxL-like motif in LHR that is individually responsible for mediating interaction with HP1.

Joppich et al. [16] have reported that similar to LHR, HP4 (Hip) binds to HP1 and HP6 (Umbrea) *in vitro*, and that these proteins colocalize at pericentric heterochromatin and form a complex *in vivo*. These authors further proposed that the association between HP1 and HP6 is mediated by heterodimerization of the chromo-shadow domain (CSD) in each protein, similar to the known homodimerization of the CSD in HP1. Structural investigation suggests that CSD dimerization results in formation of a protein-protein interaction pit that is predicted to bind one peptide per CSD dimer [29]. LHR, HP4, and HP5 each interact with both HP1 and HP6, but do not interact with each other. These interaction results combined with the limitation of CSD dimers to only bind one peptide, suggest that LHR, HP4 and HP5 are unlikely to simultaneously bind to HP1 or HP6 CSD dimers or to HP1/HP6 heterodimers. Instead, LHR, HP4, and HP5 likely interact separately with HP1 in distinct complexes.

Greil et al. [14] demonstrated that LHR (HP3) is dependent on HP1 for proper localization to heterochromatin in tissue culture cells. Here we developed an *in vivo *localization assay using YFP-tagged LHR transgenes expressed in salivary glands, in order to take advantage of the resolution available in their large polytenized nuclei. We have confirmed with this assay that HP1 is essential for proper heterochromatic localization of LHR. One potential complication of our assay is that it relies on the Gal4-UAS expression system, and may overexpress LHR compared to the wild type. The fact that LHR colocalizes with HP1 in our assay, consistent with previous reports [5,14] suggests that LHR is unlikely to be localizing outside of its normal pattern.

We also found that LHR does not depend on HP6 for heterochromatic localization in salivary glands. One possibility is that HP6 depends on LHR for localization, as suggested by Greil et al. [14] and predicted by a recent Bayesian network inference analysis based on genomic mapping studies [26]. A second possible explanation is that LHR interacts with HP6 in tissues other than third-instar larval salivary glands, and our conclusion is limited to this tissue and stage of development. A third possibility is that LHR and HP6 are differentially expressed and thus do not interact *in vivo*.

We have found that available *D. melanogaster *mutations of some of these HP genes do not suppress hybrid male lethality (Additional file 1: Supplemental Table S1). Rescue of male lethality by *Lhr*, however, requires a mutant of *D. simulans*, not *D. melanogaster*. Therefore a more comprehensive test of whether these other HP genes have any role in hybrid male lethality will require obtaining mutants in *D. simulans*.

### Conserved interactions, localization and functional properties of *Lhr *orthologs

The high rate of evolution of *Lhr *raised our interest in examining whether *Lhr *functional properties are conserved among *Drosophila *orthologs. More specifically, the previous discoveries that *Lhr *diverged between *D. melanogaster *and *D. simulans *under adaptive evolution, and that only *sim-Lhr *but not *mel-Lhr *causes hybrid lethality, strongly suggested that *Lhr *has undergone significant functional changes between these two species and possibly among other species. We found, however, that two heterochromatin-related properties of the LHR protein are conserved across species. First, LHR from seven *Drosophila *species including *D. melanogaster *interacts in yeast two-hybrid assays with two *D. melanogaster *heterochromatin proteins, HP1 and HP6, as well as with *D. simulans *HP6. Second, we found that the four LHR orthologs we tested co-localize with HP1 at heterochromatic regions when expressed in *D. melanogaster*.

We infer from these results that LHR localization to heterochromatin via binding to HP1 is both an ancestral and conserved property among *Drosophila*. From our data, we cannot exclude an alternative possibility that some LHR orthologs do not localize to heterochromatin in their native species despite doing so when expressed in *D. melanogaster*. This alternative, however, seems unlikely. First, we have shown that LHR heterochromatic localization likely depends on direct binding to HP1, and HP1 is highly conserved among *Drosophila*. Second, such a scenario would require an LHR ortholog to have evolved so as to have lost or changed protein interactions that likely occur in the ancestral state, while retaining the ability to interact with the same proteins in *D. melanogaster*.

Our data strongly suggest that LHR has not undergone a complete change of function between *D. melanogaster *and *D. simulans*, or among other Drosophila species. We therefore tested *Lhr *from four species for its ability to induce hybrid male lethality. Because we previously observed an asymmetry in the effects of *D. melanogaster *and *D. simulans Lhr *mutants on hybrid rescue, we predicted that the addition of a *D. melanogaster Lhr *transgene (and possibly other orthologs) would not complement the *D. simulans Lhr*^1 ^mutation, and thus not be lethal. Contrary to our expectation, we found that *Lhr *from both *D. melanogaster *and *D. simulans *as well as from the outgroup species *D. yakuba *suppresses *Lhr*^1 ^rescue and thus prevents rescue of lethal hybrid males (Table 2).

How do we reconcile this discovery with our previous report? We suggest that both *mel-Lhr *and *sim-Lhr *have at least some degree of hybrid lethal activity, but that *sim-Lhr *is stronger. Therefore the functional changes between *D. melanogaster *and *D. simulans Lhr *orthologs are likely quantitative and thus easily masked by gene dosage effects. The transgenic complementation experiments reported here expressed *Lhr *orthologs in hybrids in addition to that expressed from the endogenous *mel-Lhr *gene. In contrast, in our previous deletion experiments hybrids expressed only a single endogenous copy of either *mel-Lhr *or *sim-Lhr*. In addition, the Gal4-UAS system used here likely expresses *Lhr *to a level higher than in wild type animals, and it is possible that hybrid viability is more sensitive to LHR dosage than suggested by the GAL4-driven localization studies above.

What then is the functional difference between *sim-Lhr *and *mel-Lhr *that causes the asymmetry in their hybrid lethality properties? One possibility is that there is a subtle difference in their localization properties that is not detectable in our assays. mel-LHR has been reported to bind to several hundred sites along the chromosome arms, predominantly in pericentric regions [14]. A second possibility is that sim-LHR and mel-LHR have different protein interaction properties, perhaps with as-yet undiscovered partners. Finally, they may differ in effector functions such as modifying chromatin states or affecting gene regulation.

Our results with expression of LHR orthologs in foreign-species backgrounds contrast with other studies of rapidly and adaptively evolving heterochromatin proteins. One example is the centromeric protein Cid. The distantly related *D. bipectinata *Cid fails to localize to the centromere when expressed in *D. melanogaster *tissue-culture cells [30]. This study also found that conserved amino acids were essential for proper targeting of Cid to the centromere, and suggested that the rapid evolution of Cid may be a response to rapid turnover of DNA at satellites and centromeres. A second example is the OdsH protein. The hybrid sterility proteins OdsH from *D. simulans *(sim-OdsH) and *D. mauritiana *(mau-OdsH) have different localization patterns when expressed in *D. simulans *[31]. sim-OdsH associates with the X pericentric region and the 4^th ^chromosome, both repeat-rich regions of the *D. simulans *genome. Similarly, mau-OdsH localizes to the *D. simulans *X and 4^th ^chromosomes, but also to the *D. simulans *Y chromosome, and it has been proposed that this unusual localization causes decondensation of the Y leading to hybrid sterility. This change in localization is likely associated with the large number of amino-acid changes in the OdsH homeodomain between these species [32] and has been proposed to be driven by co-evolution with satellite DNAs [31].

We proposed previously that LHR also has co-evolved with rapidly evolving heterochromatic DNAs [5]. If so, this co-evolution may be indirect, since LHR does not appear to have a DNA binding domain, and its localization to heterochromatin depends on other proteins. Rapid and adaptive evolution of LHR may therefore more directly be a consequence of its interactions with other rapidly evolving heterochromatin proteins.

## Conclusions

One of the major goals in the study of speciation is to unravel the molecular bases of the genetic phenomena. Here, we have shown that despite the high levels of divergence among *Lhr *orthologs, LHR properties associated with localizing to heterochromatin and interacting with other HPs are conserved across species. In addition, we also show that the property of inducing *D. melanogaster-D. simulans *hybrid male lethality is conserved for multiple *Lhr *orthologs. This conservation of lethal activity includes *D. melanogaster Lhr*, which was previously thought to be functionally distinct from *D. simulans Lhr *in this respect. Our results suggest that the hybrid background is highly sensitive to small functional differences and that hybrid incompatibilities may result from an accumulation of subtle molecular changes.

## Methods

### Cloning and DNA manipulations

The coding sequences for *HP1*, *HP4*, *HP5*, *HP6 *and seven *Lhr *orthologs were PCR amplified with primers listed in Additional file 1: Supplemental Table S2 using Pfx DNA polymerase (Invitrogen) from the following sources: *D. melanogaster *Oregon R (for *Lhr*); *D. melanogaster w^1118 ^*(for the *HP*s and *rhino*); *D. simulans *C167.4 (for *Lhr *and *HP6*); *D. yakuba *Y9, *D. erecta *E1 and wild type strains of *D. ananassae*, *D. pseudoobscura *and *D. virilis *for other *Lhr *orthologs. All *Lhr *orthologs and *HP6 *were amplified from genomic DNA, and *HP1*, *HP4*, *HP5*, and *rhino *were amplified by RT-PCR (Invitrogen Superscript III) using 1 μg of total RNA from embryos. All coding sequences were then cloned into pENTR-D-TOPO (Invitrogen) according to the manufacturer's instructions, and verified by sequencing.

Entry vectors containing the CDS of interest were then recombined with destination vectors in an LR Clonase-mediated reaction. The destination vectors used were: pHMW for cell culture transfections, pTWV and pTWV-attB for creating Drosophila transformation vectors, (pHMW and pTWV are described at http://www.ciwemb.edu/labs/murphy/Gateway%20vectors.html, pEXP-1 for the *in vitro *binding assay (Invitrogen), and pGADT7-AD and pGBKT7-DNA-BD for the yeast two-hybrid assays (K. Ravi Ram, A. Garfinkel, and M.F. Wolfner, Cornell University; personal communication). The plasmid pTWV-attB was created by adding an *attB *site into pTWV using the primers listed in Additional file 1: Supplemental Table S2 to amplify the *attB *sequence from pTA-attB [27], followed by digestion with *Afe*I, and ligation into the *Afe*I site in pTWV. The CDS of *D. melanogaster Lhr *was recombined into pTWV to create the plasmid *pP{w^+mC ^Scer\UAS-mel\Lhr::Avic\Venus = UAS-mel\Lhr::Yfp}*. The CDSs of *Lhr *from *D. simulans*, *D. yakuba *and *D. virilis *were recombined into pTWV-attB to create the plasmids *pΦ {w^+mC ^Scer\UAS-sim\Lhr::Avic\Venus = UAS-sim\Lhr::Yfp}, pΦ {w^+mC ^Scer\UAS-yak\Lhr::Avic\Venus = UAS-yak\Lhr::Yfp}*, and *pΦ {w^+mC ^Scer\UAS-vir\Lhr::Avic\Venus = UAS-vir\Lhr::Yfp}*.

Site-directed mutagenesis of full length *D. melanogaster Lhr *was done using the QuikChange site-directed mutagenesis kit (Stratagene) by following the manufacturer's protocol with the primers listed in the primers section. The PxDxL, PxVxS and both IxPxV amino acid motifs were changed to AxAxA, and the LxVxV motif was changed to LxAxA.

### Cell culture and DNA transfections

*Drosophila *Kc-167 cells were grown at 25°C in M3 medium (HyClone) until reaching a density of 10^6 ^cells/mL. Cells were then transfected with 1 μg DNA plus 10 μL Cellfectin (Invitrogen) in 1 mL of M3 media. Cells were grown for two additional days, and then either processed, or subjected to 2 hour heat shock and 4 hour recovery, and then processed. Nuclear extracts were prepared as follows: cells were pelleted, resuspended in 1 mL Buffer 1 (10 mM Tris-Cl pH 8.0, 300 mM sucrose, 3 mM CaCl_2_, 2 mM Mg(CH_3_COO)_2_, 0.1% TritonX-100, 0.5 mM DTT) and homogenized 50 times in a dounce homogenizer (Bellco, pestle B). Cell extracts were centrifuged at 700 × g for 5 minutes at 4°C, pellets washed with 1 mL Buffer 1, and centrifuged again at 700 × g for 5 minutes at 4°C. Pellets were then resuspended in 100 μL Buffer 2 (50 mM Tris-Cl pH 8.0, 25% glycerol, 5 mM Mg(CH_3_COO)_2_, 0.1 mM EDTA, 5 mM DTT) and either used for immunoprecipitation assays or mixed with SDS sample buffer.

### Immunoprecipitation and yeast two-hybrid assays

Nuclear extracts from Kc-167 cells were mixed with mouse anti-MYC (Roche) and *in vitro *synthesized extracts were mixed with rat anti-HA (Roche) antibody and 25 μL protein-G coupled magnetic beads (Invitrogen), brought to 500 μL in IP Buffer (1 × PBS, 0.1% Triton X-100, 1 mM PMSF, 1 × protease inhibitor cocktail [Roche], 1 unit DNase [Roche], 1 unit RNase [Roche]) and rotated for 4 hours at 4°C. Magnetic bead-antibody-protein complexes were then captured following the Invitrogen Dynabeads protocol, except for the final elution, where the beads were added directly to 4 × SDS sample buffer and boiled for five minutes. Yeast two-hybrid assays were performed as previously described [5].

### Western blots

Protein samples were run on 10% SDS polyacrylamide gels and transferred to PVDF membrane (Millipore) for 60 minutes at 100 V. Blots were blocked in 5% nonfat milk for 60 minutes, rinsed with TBS-T (1 × TBS, 0.1% Tween-20) and incubated overnight at 4°C in primary antibody solution (1 × TBS-T, 5% BSA, 0.02% NaN_3_). Primary antibodies were used at 1:1000 for mouse anti-MYC (Roche), mouse anti-HP1 (monoclonal supernatant C1A9, Developmental Studies Hybridoma Bank, The University of Iowa), and rat anti-HA (Roche). Blots were washed 3 times for 10 minutes in TBS-T and incubated for 2 hours in secondary antibody solution (1 × TBS-T, 2.5% nonfat milk). Horseradish peroxidase-conjugated anti-mouse, anti-rat, and anti-rabbit secondary antibodies (Jackson Labs) were used at 1:10,000. Blots were then washed 3 times for 10 minutes in TBS-T and developed using an ECL Western Blotting kit (Pierce).

### *In vitro *binding assays

The coding sequences for HP1 and LHR-HA were cloned into the pEXP-1 vector (Invitrogen). Proteins were synthesized by mixing 1.0 μg of the appropriate plasmid DNA and 25 μL of the TnT QuickCoupled Transcription/Translation kit according to the manufacturer's protocol and incubating the mixture at 30°C for 90 minutes (Promega). 15 μL of each synthesized protein extract was then mixed with antibodies, beads, and IP buffer to 250 μL, as described above for the immunoprecipitation assay. Protein complexes were captured and immunoprecipitation was assayed by Western blot as above.

### *Drosophila *stocks and crosses

Flies were reared on standard yeast glucose media and raised at room temperature (~23°C) on a 12 hr light/12 hr dark cycle. The *D. melanogaster *mutant stock *In(1)w^m4h^; Su(var)205^5^/In(2L)Cy, In(2R)Cy, Cy^1 ^*stock was obtained from the Bloomington Stock Center. *w^1118^*; *HP6^36-5 ^*was obtained from Dr. Satoru Kobayashi [33] and *In(1)w^m4h^; Su(var)205^04^/Cy Roi *was obtained from Dr. Barbara Wakimoto. Other fly stocks are described in FlyBase [34].

*pP{UAS-mel\Lhr::Yfp} *plasmid DNA was transformed into *w^1118 ^*using P-element mediated transformation. The *P{UAS-mel\Lhr::Yfp}1 *and *P{UAS-mel\Lhr::Yfp}2 *transgene insertions are on chromosome 2 and *P{UAS-mel\Lhr::Yfp}3 *is on chromosome 3. The 3 different *pΦ {UAS-Lhr::Yfp*} plasmids were recombined into an *attP *site on chromosome 3 by injection into the strain *y^1 ^M{vas-int.Dm}ZH-2A w; M{3xP3-RFP.attP}ZH-86Fb *[35] by Genetic Services (Cambridge, MA) using the *Φ *C31 site-specific integration method [27]. For the LHR localization assays in mutant HP backgrounds, *P{UAS-mel\Lhr::Yfp}3 *was recombined onto a chromosome containing the salivary gland specific GAL4 driver *P{GawB}c729*. The recombinant chromosome 3 was then crossed into mutants for *HP1 *and *HP6*. For the LHR localization assays in an otherwise wild type background (both whole-mount and squashed polytene analyses), F1 larvae heterozygous for *P{UAS-mel\Lhr::Yfp}3 *or one of the 3 different *Φ {UAS-Lhr::Yfp*} transgenes and *P{GawB}c729 *were analyzed. The *P{Act5C-GAL4}17bFO1 *transgene was used as a source of GAL4 in the *Lhr^1 ^*complementation crosses.

### Immunofluoresence

For whole-mount salivary gland preparations, tissues from third instar larvae were dissected in PTX (1 × PBS, 0.3% Triton X-100), glands were fixed for 8 minutes in 3.5% paraformaldehyde in PTX and rinsed 3 times in PTX. During the third wash, DAPI was added to a concentration of 1 μg/ml and samples were incubated for 2 minutes at room temperature. Glands were then washed 3 × in PTX for 5 minutes and mounted in Vectashield.

For polytene squashes, glands were dissected in 0.7% NaCl, fixed for 8 minutes with 1.85% paraformaldehyde in 45% acetic acid, and squashed. Chromosomes were incubated overnight at 4°C with the primary antibodies mouse anti-HP1 (DSHB) at 1:10 and rabbit anti-GFP (Invitrogen) at 1:100 in PBS + 0.05% Tween-20, followed by incubation for two hours at room temperature with Alexa anti-mouse 546 and Alexa anti-rabbit 488 secondary antibodies (Invitrogen) at 1:300 in PBS + 0.05% Tween-20. All samples were mounted in Vectashield.

### Divergence Calculations

Orthologous coding sequences for each gene were obtained from Flybase [34] aligned using ClustalW [36], and processed into. MEG format using MEGA [37]. Divergence data for nucleotide changes were calculated with DnaSP [38].

## Authors' contributions

NB and DB designed the experiments. NB performed the experiments and collected the data. NB and DB analyzed the data and wrote the manuscript. All authors read and approved the final manuscript.

## Supplementary Material

Additional file 1 **Supplementary Figures and Tables**. Supplementary Figures S1-S5 and Tables S1-S2 are available in PDF format. Click here for file
