# Supplementary material for: Functional conservation of the Drosophila hybrid incompatibility gene Lhr
Source: BMC Evol Biol. 2011 Mar 2;11:57. doi: 10.1186/1471-2148-11-57 (PMC3060119; doi:10.1186/1471-2148-11-57)
Supplement: Additional file 1 — Supplementary Figures and Tables. Supplementary Figures S1-S5 and Tables S1-S2 are available in PDF format. [file 1471-2148-11-57-S1.PDF]

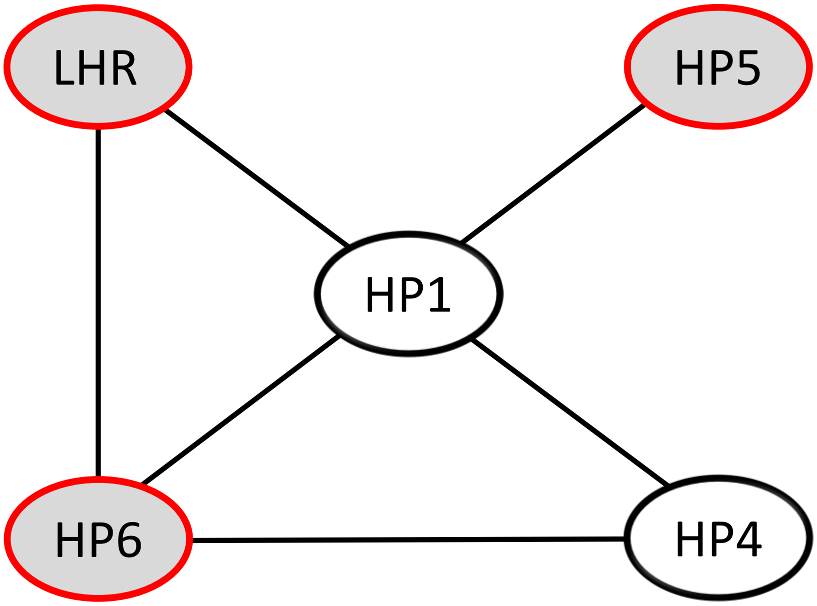

Figure S1 - Diagram of interaction and molecular evolution data of five HPs.
Solid lines connect the proteins that have been reported to interact in previous studies [1-3]. The proteins have been grouped based on data in Table 1 into rapidly evolving (K_A/K_S>0.7; red border, shaded) and non-rapidly evolving (K_A/K_S <0.2; black border, not shaded).

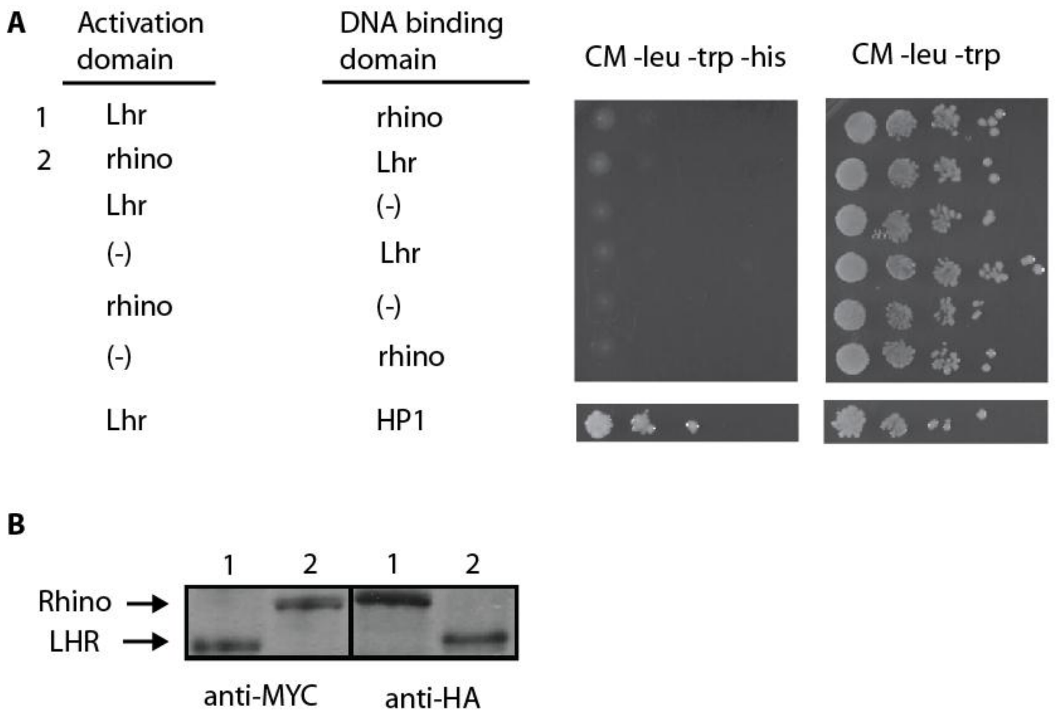

Figure S2 - No interaction was detected between LHR and the HP1 paralog Rhino by yeast two hybrid analysis.

Interactions were analyzed in yeast two-hybrid by activation of HIS3 and growth on complete media lacking histidine (CM -trp -leu –his); growth controls (CM -trp –leu) contain histidine. A) No growth is seen for both LHR-Rhino combinations; indicting that the two proteins do not interact. B) Genotypes 1 and 2 from (A) were tested by Western blot in order to demonstrate that both fusion proteins are expressed in yeast.

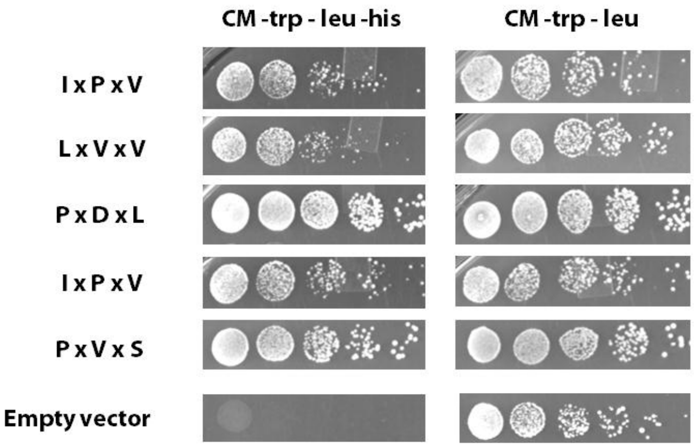

Figure S3 - Testing if five PxVxL variants in LHR mediate the interaction with HP1 in a yeast two-hybrid assay.

Independent mutagenesis of each PxVxL variant in full length LHR failed to abolish interaction between LHR and HP1, indicating none are individually necessary to mediate this interaction. Growth in the (CM -trp –leu –his) column indicates an interaction, and growth in the (CM -trp –leu) column indicates both the activation domain and DNA binding domain plasmids are present.

A) HP1 chromo-shadow domain

```
D.melanogaster      GLEAEKILGASDNNGRLTFLIQFKGVDQAEMVPSSVANEKIPRMVIHFYEERLSWYSDNED
D.simulans          GLEAEKILGASDNNGRLTFLIQFKGVDQAEMVPSSVANEKIPRMVIHFYEERLSWYSDNED
D.yakuba            GLEAEKILGASDNNGRLTFLIQFKGVDQAEMVPSSVANQKIPQMVIHFYEERLSWYSDNED
D.erecta            GLEAEKILGASDNNGRLTFLIQFKGVDQAEMVPSSVANQKIPQMVIHFYEERLSWYSDNED
D.ananassae         GLEAEKILGASDNNGRLTFLIQFKGVDQAEMVPSTVANVKIPQMVIHFYEERLSWYSDNED
D.pseudoobscura     GQEAEKILGASDNNGRLTFLIQFKGVDQAEMVPSSIANLKIPQMVIGFYEERLSWYSDNED
D.virilis           GLEAEKILGASDNNGRLTFLIQFKGVDQAEMVPSTVANVKIPQMVIRFYEERLSWYSDNED
Clustal Consensus   * ********************************::** ***:*** **************
```

B) LHR C-terminus

```
                    ....|....|....|....|....|....|....|....|....|....|....|....|....|....|....|....|
D.melanogaster      ---FLLSQLPFVKSMAQAERRHLEVEVLALILEQERQEKATRKLGPKDLETVQSEYDEF----------LKAIRVKELPA
D.simulans          ---FLLSQLPFVKSLSPAERRHLEVEVLDIILEQERQEKATRQLGPMELKTVQSEYEEF----------LKAIRVKELPA
D.yakuba            ----------FLKSLSQSERRRLEVEVLDLILEQERLEKATQELGPKELETAQSEYDEF----------LKAIRVKELPA
D.erecta            ---FLLSQLNLVKSLSQSGRRRLEVEVLDLILEQERLEKATQKLGPKELETAQSEYDEF----------LKAIRVKELPA
D.ananassae         ----------FLKTIR--------VKELPNNILT-QIIPKVDPSNKMGLVITQVFSTSS----------KEEIPSTSKAK
D.pseudoobscura     FLGSVRVKELPISALAQLSLDGPQYKPAQPKIAHTFAIPGPRPKKKRVISSVSGGQDLINIPTATVSIPIQDFKSNVVGP
D.virilis           ----------FLKAIR--------VKELPVQVSSRRSSASSNGSSKPDSSAAARSSNLV----------ISDVRGHSEPQ
Clustal Consensus              :.::          :     :                   .                  . .

                                   90       100       110       120       130       140       150       160
                    ....|....|....|....|....|....|....|....|....|....|....|....|....|....|....|....|
D.melanogaster      DNLLSPAMDRFGISPHKIRPNVASANKR-------IRYNNACKGSNDVKIKIETAIEPKIKDTTKC-------------D
D.simulans          NTLLSPAIDGFRISPRNIRPNLASANKR-------ISYNGVSNESNYVKIKTETAIEPKIEDATKF-------------D
D.yakuba            DTLIRSTIDGFRISPHSN-PSIINANKRSVGSSRTISDNGISNGSNDVEIKTEKPDEPENKDANKF-------------D
D.erecta            DTLLRPAMYRFRISPHSK-QNIVNANGRNAGTSKTISDNYVSNGSNDVEIKTEKADEPVTQGKKKF-------------D
D.ananassae         VKPE--PIESPVRS--------------------------------QKPKESPASGSRSSSRKST-------------Q
D.pseudoobscura     IDPAPAVSPPLDINVIGAKKEPVECPPSVSPPLDIKTEVEVALAAIELAIEMENVRENKPEEAQQKSPEKNGTPVEKPDD
D.virilis           PEPANPPTSPLVIK--------------------------------NEPPDQLEIELQPLSKQSP-------------D
Clustal Consensus                .                                      .          .              :

                            170       180
                    ....|....|....|....|....|
D.melanogaster      ERPIETPRFVPLKSAKYYIKRCRI-
D.simulans          ERPIETPRYVPLKSAKYYIKSCRI-
D.yakuba            KRPTETPRYVPLKSAKYYIKSCRI-
D.erecta            DRPTETPRYVPLKSAKYYIKSCRI-
D.ananassae         KTASS-PQFVPIKSAKYYIKPVRV-
D.pseudoobscura     ATLNPNPRYIPIKSAKYYTKKLIV-
D.virilis           KEADPRLRYVPLKSAKYYVKKVQVR
Clustal Consensus          :::*:****** *   :
```

Figure S4 - The CSD of HP1 is highly conserved in contrast to the C-terminus of LHR. Alignment of the domains in (A) HP1 and (B) LHR found to mediate the interaction in *D. melanogaster*, from seven Drosophila species.

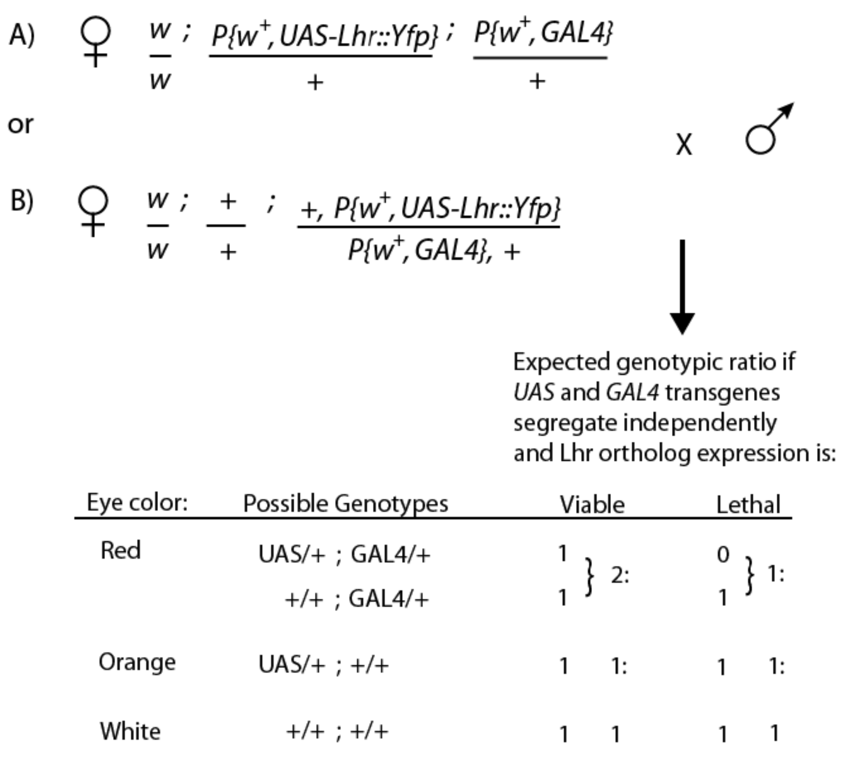

Figure S5 - Complementation crosses to test for suppression of hybrid male rescue.

D. melanogaster female parents heterozygous for *GAL4* and *UAS-Lhr-Yfp* transgenes on different chromosomes (A) or the same chromosome (B), were crossed to *D. melanogaster* (control) or *D. simulans* Lhr^1 males (Table 2). The transgene insertions *P{UAS-mel\Lhr::Yfp}1* and *P{UAS-mel\Lhr::Yfp}2* were used in scheme (A). In this scheme the UAS and GAL4 transgenes segregate independently. The *P{UAS-mel\Lhr::Yfp}3* transgene insertion and the *D. simulans*, *D. yakuba*, and *D. virilis Lhr-Yfp* transgenes inserted into cytological position 86Fb were used in scheme (B). In this scheme the transgenes will segregate independently if they are unlinked on chromosome 3. Both the *GAL4* and *UAS-Lhr-Yfp* transgenes are marked with w^+, and the *GAL4*-containing transformant has a darker eye color and is epistatic to the lighter *UAS*-containing transformants. Therefore, the red-eye class of progeny could potentially be composed of two different genotypes.

Table S1 - HP mutations do not dominantly suppress hybrid male lethality

Female parent[a]	F1 male progeny		F1 female progeny	
	+/-	+/+	+/-	+/+
HP1[b]	0	0	151 [n.s.]	145
HP4[c]	0	0	168 [n.s.]	176
HP5[d]	0	0	202 [n.s.]	184
HP6[e]	0	0	191 [n.s.]	178

[a]– *D. melanogaster* virgin females were crossed to *D. simulans* w^{501} males and the F1 progeny were scored for the mutant (+/-) or balancer (+/+) chromosome. Crosses were performed at room temperature (~22-24°). Full genotypes of *D. melanogaster* female parents are as follows:

[b] *Su(var)2-5*^{02}*/Bc Gla*

[c] y^1 w^{67c23}; $P\{w^{+mC}\ y^{+mDint2}$=EPgy2} $HP4^{EY01733}$/TM3, Sb^1 Ser^1

[d] y^1 w^{67c23}; $P\{w^{+mC}\ y^{+mDint2}$=EPgy2} $HP5^{EY10901}$/FM7i, Act-Gfp

[e] w^{1118}; $HP6^{36\text{-}5}$/Bc Gla.

n.s. – The observed number of F1 females inheriting the *HP* mutant chromosome was not significantly different than the expected value by Chi-square analysis ($p > 0.05$).

Table S2 - Primers

attB
F-5’-AAACCCAGGCCTATGCCCGCCGTGACCGTC-3’
R-5’-AAACCCAGGCCTGATGTAGGTCACGGTCTCG-3’

D. melanogaster Lhr
F- 5’-ATGAGTACCGACAGCGCCGAGGAA-3’
R- 5’-TCATGTTCTCAGCGTAGGCCG-3’

D. simulans Lhr
F- 5’-ATGAGTACCGACAGCGCCGAGGAA-3’
R- 5’-TCATGTTCTCAGCGTAGGCCG-3’

D. yakuba Lhr
F- 5’-ATGAGTACCGACAGCGCCGAGGAA-3’
R- 5’-TCATGTTCTTAGCGTAGGCCTTCTG-3’

D. erecta Lhr
F- 5’-ATGAGTACCGACAGCGCCGAGGAA-3’
R- 5’-TCATGTTCTCAGCGTAGGCCGCC-3’

D. ananassae Lhr
F- 5’-ATGAGCGAGGAAGTTGGGGCTCTT-3’
R- 5’-TCAATCTTTTTGTGAATGGCGACT-3’

D. pseudoobscura Lhr
F- 5’-ATGGATACCGAAGCGGGAATGGAC-3’
R- 5’-TTAACGTTTCTTTGGGCGCTTTGC-3’

D. virilis Lhr
F- 5’-ATGGATGGCTTGGAGGATGCATCC-3’
R- 5’-TCAGGAGCTTCTGTTGCGGGTCTT-3’

D. melanogaster Su(var)2-5
F- 5’-ATGGGCAAGAAAATCGACAACC-3’
R- 5’-TTAATCTTCATTATCAGAGTACC-3’

D. melanogaster HP4
F- 5’-ATGTCACCGAAGACTAAAAAAATG-3’
R- 5’-GATTGTCAGATTAGACTCC-3’

D. melanogaster HP5
F- 5’–ATGGATATTTTTGATATGG-3’
R- 5’-CGATTCTAACGCGGTCATTATTTC-3’

D. melanogaster HP6
F- 5’-ATGCCCAGCTCCACTTTGACG-3’
R- 5’-CTAGGCATTTCGTGATCGTTTCTTC-3’

D. simulans HP6
F- 5’-ATGGATCCGACGAAGTCTAAGACC-3’
R- 5’- CTAGGCACTTGGTGCCCG-3’

D. melanogaster Rhino
F- 5’-ATGTCTCGCAACCATCAGC-3’
R- 5’-TTACTTGGGCACAATGATCCTC-3’

IxPxV #1 to AxAxA
F-5’-GGTATCCCTTCCTTGCCATCGCGCAGGCGGAGCCCAAAATGGAC-3’
R-5’-GTCCATTTTGGGCTCCGCCTGCGCGATGGCAAGGAAGGGATACC-3’

LxVxV to LxAxA
F-5’-CCGGCATTTAGAAGCGGAGGCCCTCGCCCTCAT-3’
R-5’-ATGAGGGCGAGGGCCTCCGCTTCTAAATGCCGG-3’

IxPxV #2 to AxAxA
F-5’GCATTAGCCCACACAAAGCAAGAGCCAATGCCGCAAGTGCCAATAAAAG-3’
R-5’CTTTTATTGGCACTTGCGGCATTGGCTCTTGCTTTGTGTGGGCTAATGCC-3’

PxDxL to AxAxA
F-5’-GGTGAAAGAGCTCGCCGCAGCCAATGCCCTCAGTCCGGCC-3’
R-5’-GGCCGGACTGAGGGCATTGGCTGCGGCGAGCTCTTTCACC-3’

PxVxS to AxAxA
F-5’-CCACACAAAATAAGAGCCAATGCCGCAGCCGCCAATAAAAGAATC-3’ R-5’-GATTCTTTTATTGGCGGCTGCGGCATTGGCTCTTATTTTGTGTGG-3’

Supplementary References

1. Giot L, Bader JS, Brouwer C, Chaudhuri A, Kuang B, Li Y, Hao YL, Ooi CE, Godwin B, Vitols E *et al*: **A protein interaction map of Drosophila melanogaster**. *Science* 2003, **302**(5651):1727-1736.
2. Brideau NJ, Flores HA, Wang J, Maheshwari S, Wang X, Barbash DA: **Two Dobzhansky-Muller genes interact to cause hybrid lethality in Drosophila**. *Science* 2006, **314**(5803):1292-1295.
3. Joppich C, Scholz S, Korge G, Schwendemann A: **Umbrea, a chromo shadow domain protein in Drosophila melanogaster heterochromatin, interacts with Hip, HP1 and HOAP**. *Chromosome Res* 2009, **17**(1):19-36.
